# A qualitative analysis of the coping reservoir model of pre-clinical medical student well-being: human connection as making it ‘worth it’

**DOI:** 10.1186/s12909-020-02067-8

**Published:** 2020-05-19

**Authors:** Kelly Rhea MacArthur, Jonathon Sikorski

**Affiliations:** 1 Department of Sociology and Anthropology, University of Nebraska Omaha, 6001 Dodge St, Omaha, NE 68182 USA; 2 Department of Psychiatry, University of Nebraska Medical Center, 985575 Nebraska Medical Center, Omaha, NE 68198 USA

**Keywords:** Pre-clinical medical students, Burnout, Distress, Wellness, Optimism, Hope, Coping, Doctor-patient relationship, Curriculum, Qualitative research methods

## Abstract

**Background:**

By all indications, well-being among physicians is poor, which manifests in various outcomes, including burnout, depression/anxiety, low life satisfaction, alcohol/substance misuse, suicide ideation, and suicide. Despite the vast literature on physician burnout, there is relatively little research on how pre-clinical experiences in medical school may be an antecedent to subsequent poor health among physicians. Here we focus on two neglected areas within the literature by focusing on the pre-clinical stage of medical school and the positive, as opposed to exclusively the negative, aspects of the medical school experience as it affects well-being.

**Methods:**

This study utilizes the metaphor of the Coping Reservoir Model as a theoretical and analytical framework for understanding medical student well-being by identify the ‘depleting’ and ‘replenishing’ inputs that are deposited into students’ coping reservoirs. We analyze 105 medical students’ reflective writings using a data analytic process consistent with an interpretive description approach, engaging in a hierarchical 3-step coding process to identify the main replenishing inputs deposited into students’ coping reservoirs.

**Results:**

The main depleting inputs that we identify are consistent with those identified by The Coping Reservoir Model. In addressing our main research question regarding the replenishing inputs, results show the main positive factors are psycho-social resources, intellectual stimulation, and social support/relationships. Most importantly, relationships with patients shape all three of these positive factors and provide the main source of hope that the stress of medical school will get better.

**Conclusions:**

What allows students to frame their experiences with hope and optimism are the connections they form with each other and with patients. The prolonged stress of medical school is made “worth it” in hopes that it will “get better” with more meaningful patient interaction in the future. These results that emphasize the positive aspects of medical school are discussed in context of their theoretical contributions to The Coping Reservoir Model and the practical implications for medical education to improve medical student well-being by facilitating human connection.

## Background

By all indications, well-being among medical students is poor, as studies consistently show they have poor well-being that manifest in various forms, including burnout [1], depression/anxiety [2–6], low life satisfaction [7], alcohol/substance misuse [8], suicide ideation [9], and suicide [10]. In fact, one study of seven U.S. medical schools found that approximately 82% of medical students reported at least one form of distress, all seven of which were significantly associated with suicide ideation [3]. Exacerbating the prolonged stress that characterizes medical school is that the adverse effects of stressors are not mitigated by health-promoting behaviors, as medical students tend to engage in unhealthy behaviors regarding diet, caffeine, nicotine, alcohol/drugs, physical exercise, and sleep [11]. They also face barriers to seeking mental health treatment, most notably stigma [12]. With physicians dying by suicide at a rate nearly twice that of the general population [10] and higher than any other occupational group [6, 13], more attention needs to be paid to the early risk factors of poor well-being among medical students.

The evidence suggests that poor well-being among physicians originates in medical school. Studies show that the health of medical students begins on par with their non-medical student peers and then diverges from there. In fact, at all stages of medical school, medical students experience far worse health than their non-medical student counterparts [2, 14], as longitudinal analyses show that medical students’ well-being grows significantly worse throughout medical school, into residencies, and beyond [4, 7, 14–16]. These studies suggest that it is not individual psyches or the life stage of young adulthood that is the problem. Rather, the process of medical school itself seems to be putting students on a trajectory of progressively poor well-being. The purpose of this study is to examine the risk and protective factors at the beginning of this process, the first year of medical school, in order to ultimately prevent students from getting on a track of poor health.

### The coping reservoir model

As first year students adjust to the academic demands of medical school, the degree to which exposure to stressors adversely affects well-being can be buffered by effective coping strategies—what the literature that focuses on burnout tends to term “resiliency,”— generally defined as positive adaptations to adversity [17]. Studies show, for example, that students enter medical school with health-protective social-psychological resources, such as optimism, acceptance, joy, and positive reframing [18–20] and such positive attitudes are associated with better well-being [18, 21]. Unfortunately, most medical students are not able to maintain a positive attitude throughout the first year or beyond [19, 20].

To identify how medical students cope with the vast stressors of the first year of medical school that affect their well-being, Dunn and colleagues proposed a model in which they conceptualize medical student well-being as a dynamic process in which both positive and negative factors are constantly being inputted into students’ metaphorical coping reservoirs [22]. Whether burnout or resiliency result depends on the strength of this reservoir as affected by these replenishing and depleting factors, which is also affected by the internal structure of the reservoir itself, including students’ personality traits, temperament, and coping style, such as obsessive-compulsiveness. The main negative inputs are stressors, mostly from perceptions about the structure of the curriculum, but also from personal life events; internal conflict defined as doubts about one’s decision to become a doctor; and competing demands for their time and energy. The main factors that replenish students’ reservoirs are hypothesized to be social support from family and friends; social activities; mentorship from faculty and peers; and intellectual stimulation. Dunn et al. theorize that there is an internal structure of the reservoir itself, which includes students’ personality traits, temperament, and coping style, such as obsessive-compulsiveness [22]. Most other studies rely exclusively on the assumption that the goal should be to identify how to cope with stress, such as with physical exercise, positive reframing, and seeking mental health care [23]. Several studies examine the relative efficacy of different types of coping among first year medical students, with problem-solving and ‘active’ coping mechanisms tending to be more effective than avoidant coping [24, 25]. The Coping Reservoir Model, in contrast, is unique in that it recognizes that there are positive aspects of medical school aside from coping mechanisms that mitigate the adverse effects on well-being that the negative stressors can have.

While there have been no direct empirical tests of the full model, several studies rely on the overall paradigm of the coping reservoir as useful for understanding medical student well-being [4, 18, 26, 27], although research on the arguably critical time period of the first year of medical school is limited. The main goal of this study is to test the utility of the coping reservoir model to understand medical students’ well-being by identifying the depleting and replenishing inputs that are deposited into preclinical medical students’ coping reservoirs. In examining whether the model is appropriate for assessing pre-clinical medical students’ well-being, we analyze medical students’ reflective writings to address the following research question: *What are the main replenishing inputs into medical students’ reservoirs?* This study will contribute to the literature that seeks to improve medical student well-being by not limiting the analysis to the negative inputs into students’ reservoirs.

## Methods

### Setting & data collection

This study is a qualitative analysis of preclinical medical students’ reflective writings that were completed as part of a wellness curriculum during Phase I of their training at a Midwestern American medical school. The medical education curriculum occurs in three Phases, including a pre-clinical stage in the first 18 months, clinical rotations in the next 12 months, and individualized training in one’s specialty choice in the last 13 months. The wellness coil occurred during Phase I and the students in this sample are in their 15th month of Phase I, which integrates basic, clinical, and health systems sciences, organized by organs systems of the body. The general goal of the wellness “coil” is to familiarize medical students with a wide variety of wellness and stress management strategies. The wellness coil occurred during the first 11 blocks of medical school over the course of 18 months, roughly once every six weeks, and was broken into two parts: an e-module (50 min) and live lecture (50 min), although, since students were not required to attend, live lectures were recorded and posted on the online course management page. Lectures averaged between 45 and 75 students attending in person. Lectures were linked to the broad topics within the concurrent medical education block. For example, exercise was discussed during the muscle, skeletal, and kinesiology block; nutrition during the renal block, and so on. Each live lecture began with a brief discussion by either the course facilitator or medical faculty member, which reviewed how the lecturer implemented wellness strategies in their personal lives and to review or demonstrate a wellness strategy. This time also allowed course facilitators to check in with students about their stress and remind them of available resources and supports. This short discussion was then followed by students engaging in reflective writing in response to a given prompt.

Of the 11 class meetings, the reflective writing completed during the 10th meeting in September of their second year was chosen as the focus of this study. This prompt asked students to “write a story about a frustrating experience during medical school” and “a story about a rewarding experience during medical school.” After given the prompt, students were verbally instructed that they could choose either a frustrating or rewarding experience and they had 7 min to complete the writing, although they had until midnight that night to submit the writing on the online course management website. In-class writing was then followed by a small group discussion in which students were asked to read aloud their reflections and engage in discussion with other students. Students and facilitators were directed to not talk to the author directly, but to rather discuss the writing (e.g., style, content, themes, etc.). The students were not allowed to provide context to their writing before sharing as this often derailed conversations or usurped the discussion. During the discussions, students were encouraged to use person-first language – “I noticed that the author explained …” , and facilitators were directly instructed to neither validate (“I remember struggling with …” ) or invalidate (“It seems that way but it really isn’t …” ). The positive or negative story that came to mind when asked to choose from a year’s worth of experience is arguably telling in and of itself. Because of the broad nature of the writing prompt, asking for stories, and the timing of that class period—the analysis here represents what is important *to them*, *in their own words* in regards to the frustrating and rewarding aspects of medical school.

### Study participants

One-hundred and thirty-one medical students were enrolled in the wellness coil in August 2017 and, of these 131 who were invited to participate in study, 13 did not consent, 6 students did not continue with the program in the year between obtaining consent and the writing of the prompt analyzed here, and 7 did not submit a writing for the prompt that was analyzed for this study. There were a remaining 105 medical students included in this analysis constituting an approximate response rate of 89.7%. All de-identified writings were uploaded for analysis into Dedoose 8.1.8. (SocioCultural Research Consultants, UCLA), a web-based, mixed methods data analysis program. All study procedures were approved by the Institutional Review Board (522–17-Ex).

### Data analysis

The authors followed a process consistent with an interpretive description approach to qualitative data analysis, in which we engaged in a hierarchical 3-step coding process, beginning with open coding, then moving on to axial coding, and completing the process with selective coding [28]. Specifically, we analyzed the data from January to March 2019, beginning with doing an initial read-through of all 105 reflective writings, using open, line-by-line coding methods. We then used axial coding to refine those broad themes by relating coding categories to subcategories. After axial coding of about half (60) of the reflective writings, the next stage consisted of engaging in a consensus-based process in which we had regular discussions, condensing and adjusting codes, until all codes were agreed upon. We then selectively re-coded the original 60 writings, as well as the remaining 45 reflective writings, by, on the one hand, explicitly applying Dunn and colleagues’ metaphorical concept of a coping reservoir [22], while on the other hand trying to detect the nuances of depleting and replenishing factors. That is, we coded for the 3 depleting and 4 replenishing factors included in the original Coping Reservoir as discussed above while also line-by-line open-coding for additional themes and sub-themes.

Consistent with the suggestions for qualitative research on health professions education [29], we conducted a member check in which an email was sent to all students in the class. The two that agreed to participate in the member check were given a table with a description of all of the themes and a sample quote and were asked whether 1) the themes resonated with their experiences; 2) the chosen quotes were an accurate representation of the themes; and 3) any themes seemed to be missing. While no substantive changes were made to the analysis as a result of the member check, the process and participant feedback increased the validity of our interpretations of the reflective writings and the identification of the main positive and negative medical school experiences. Additionally, one of the comments about the theme of peer social support prompted the authors to go through all 105 of the reflective essays and specifically look for disconfirming cases of all themes. In the end, this coding and data analysis process adheres to all twenty-one Standards for the Reporting Qualitative Research (SRQR) set forth by Academic Medicine [30].

## Results

### Overall findings

As Table 1 shows, this sample of medical students generally focused on the negative aspects of their first year, or at the very least, the negative experiences were more prone to recall. An overwhelming majority (73.33%) emphasized the negative, either by writing solely about a frustrating experience or by writing about both a frustrating and rewarding experience, but frustrating first. Nonetheless, less than half (41.9%) chose to write about a negative experience only, suggesting that, for a majority of them, the positive aspects are also relevant for determining how they assess their first year of medical school. Their choice of topic is just one indication, and a crude of measure, of whether students tend to focus on the frustrating experiences. In contrast, results of the analyses below provide a more inductive, interpretive approach to identifying the nature of the replenishing factors. In alignment with our research question, below we first list the main negative inputs into pre-clinical medical students’ coping reservoirs and then discuss the main replenishing factors in detail.

**Table 1:** Positive or negative emphasis of writing choice (*N* = 105)

*Topic Choice*	*N (%)*
Frustrating experience only	44 (41.9%)
Both: Frustrating experience first	33 (31.43%)
**Total Negative Emphasis**	**77 (73.33%)**
Rewarding experience only	5 (4.76%)
Both: Rewarding experience first	18 (17.14%)
**Total Positive Emphasis**	**23 (21.90%)**
Neither/Can’t tell	5 (4.76%)
**Total**	**105 (99.99%)**^a^

^a^Total is slightly off from 100 due to rounding

### Main depleting factors

Our analysis revealed two main factors that deplete from pre-clinical medical students’ coping reservoirs. First, to borrow Dunn and colleagues’ terminology of *curricular stress* [22], we find that the main negative aspects of the first year of medical school are due to students’ perceptions of the structure of the curriculum, including course requirements, lectures, the amount of material, the scheduling of exams, grading policies, and the faculties’ teaching methods. Second, students’ sources of stress largely stem from the ways in which they think about, or psychologically frame, their medical school experiences, here called *social psychological barriers*. Within these two main categories of depleting factors of psychological barriers, we identified several sub-themes, including feelings of loneliness, imposter syndrome, and internal conflict about being a doctor. In addition to the ones listed here, several other less salient stressors were identified, which are available from the authors upon request.

### Main replenishing inputs

**Fig. 1:**
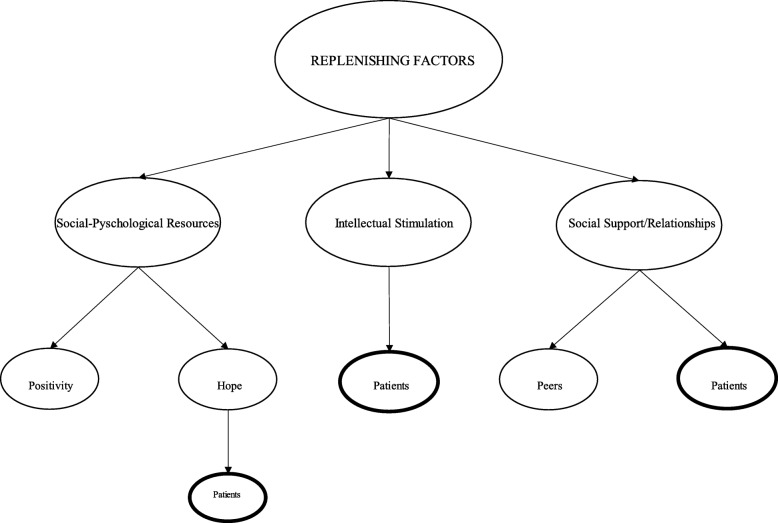
Thematic Map of Replenishing Factors

Our research question asked what are replenishing inputs into pre-clinical medical students’ coping reservoirs? As shown in the thematic map depicted in Fig. 1, we identified three broad themes of positive factors, with several sub-themes within each. First, *social psychological resources*, or students’ abilities to remain positive and have hope in the face of a great deal of stress, help to keep them going, with the main source of hope being based in idealizations of future relationships with patients. Second, what Dunn and colleagues call *intellectual stimulation* also refills students’ reservoirs and help them cope with the demands of medical school [22]. We find that, the majority of the time, intellectual stimulation is based on various types of interactions with patients, so we label the only subtheme under this category *patients.* Third, students were replenished by meaningful human connection*,* mostly with their medical student peers, but also with their families, friends, partners, and patients, what we call *social support/relationships*.

#### Social psychological resources: light at the end of the tunnel

Despite students’ extreme negativity that manifests mostly in the form of self-doubt, medical students also demonstrated a remarkable degree of idealism, or what can be considered *social psychological resources*. Some students consciously made concerted attempts to think *positively* and let things “roll off my back” (Med Student #40), or “focus on the bigger picture” (Med Student #115), such as Med Student #15:

“Every time I find myself getting down or frustrated about something that is out of my control and just part of being a medical student, I try to focus on the positive ways that medical school has changed my life.”

Other students, while not explicitly recognizing that they are trying to be optimistic, were clearly and actively trying to do so, such as Med Student #76 who focused on how things were getting better after the passing of his grandfather:

“I can finally say I am starting to get my life and school together. I’m thriving in school, spending time with friends, and enjoying life and medical school. This past year was the hardest of my life, but the frustration of life and medical school is turning into happiness one day at a time.”

And, Med Student #2 similarly remarked how, albeit frustrating, it is important to have a positive attitude toward medical school:

“[Medical school] has been incredibly frustrating but has also taught me a few important lessons. Don’t stress about things you cannot control because it will only hurt you and your mental resilience. Focus on the good. There will always be things that are hard. Remember to laugh and prioritize what you love.”

Related to students’ positivity and idealism is a sense that it is going to get better.

This social psychological resource of *hope* buffers students’ stress by allowing them to view it as a “stepping stone to becoming a doctor” (Med Student #97), to the brighter future when they ultimately will be practicing medicine.

When expressing optimism, this hope is often based on interacting, and developing relationships, with future patients. The following student perfectly summarized how the focal point of students’ hope is the human connection with patients they will have once they start practicing medicine. Reflecting upon a perceived embarrassing situation in which he forgot the function of a pharmaceutical while shadowing his preceptor, he then concluded:

“Then I realized something, what I love about medical school is yet to come. I didn’t come to medical school to regurgitate facts, play memorization games, or try to outcompete my peers on A-D exams that asks me questions such as, what type of enzyme is B-amylase. To tell you the truth I really don’t care what type of enzyme B-amylase is, that’s something I’ll google. What I love about medical school is this idea in my mind that hopefully someday soon I will be working with real people, not just the fake ones I read about in vignettes. Shaking their hands, smiling, treating, and in some cases grieving. What I love about medical school is the idea that one day I’ll be dealing with the issues of humanity and that no matter how small I may be, I will have some small part to play in others lives while I’m here.” – Med Student #36.

Like Med Student #36 did, other students also had the ability to hold out hope, as Med Student #41 did when he saw ‘the light at the end of the tunnel” after being permitted to interact with surgical patients during his three-week rural surgery care block. This hope, which stems from idealistic notions of the doctor-patient relationship, is a social-psychological resource in that it likely buffers the potentially adverse effects of stress and prevents students from leaving medicine—at least temporarily. Med Student #66 expressed this sentiment rather prolifically:

“The thought of the future, caring for people as a physician, drives me every day. It fuels me through the frustrations and challenges. Even the slightest glimpses of the future ignites my fire.”

#### Patients: Silver Linings & Necessary Reminders

In addition to future patients being a source of hope, we identified three other, non-mutually exclusive, ways in which patients served as replenishing inputs and a mechanism to: 1) apply their medical knowledge; 2) help people; and 3) develop meaningful relationships. In fact, almost any time they spoke of something positive (other than grades), it was related to patients in one way or another, even with standardized patients or shadowing a preceptor. Meaningful encounters over time where, for example, they had the opportunity to witness patients recover surely inspired them, but any clinical experience was enough to serve as a *necessary reminder* of why they decided to be a doctor and why they were suffering through extreme stress. In short, many students in this sample said that patients—including visions of their future patients— made medical school “worth it.”

Students often contrasted clinical experiences with “real” people to all of the lectures and exams, such as when Med Student #45 remarked:

“Studying lecture material all the time can become overwhelming and monotonous, but being able to use that knowledge to help solve a patient’s problem gave me solace in the hard life of a medical student. Reminders such as these become extremely comforting in times of distress.”

Students also found meaning and reassurance of any internal conflict about becoming a doctor when they were able to help family members with medical problems, shadowing in clinics, and volunteering. For example, one student described his most rewarding experience as *not* seeing his “buddy” one day in the hospital when volunteering because the patient had been allowed to go back to third grade after having been under medical supervision for three years (Med Student #70). Another student expressed gratitude for being a small part of a man’s journey to recovery and exclaimed that this is “why I love medicine” (Med Student #53). Med Student #79 was similarly reminded of his passion for medicine through experiences with patients:

“Working with my preceptor has been rewarding because it has provided rationale for why I do what I do every day. The reality of what we are striving for as medical students can easily be diluted by our every day regimen. I often have to remind myself why I am going to class (or not) and study until long after the sun goes down every day. Seeing patients in clinic with my preceptor has been a tangible reminder—and arguably a justification—for why I continue to live the life of a medical student.”

#### Social support/relationships: becoming family

The third replenishing factor that this analysis illuminates is the importance of meaningful social relationships, or human connection. Although they also talked about the importance of old friends, family, and partners when discussing the value of relationships, students most appreciated the social support from, and the connections they made with, their medical school peers. Med Student #74 explained how the best part of medical school is the new friendships:

“A wonderful experience I have had since starting medical school would be the interactions I have had with people. I remember interviewing to be accepted into medical school, and we sat around and discussed what medical school was like with a third year student. He discussed that his class was really close and supportive and recommended that we aim for the same, as it was less stressful than being a super competitive class where you don’t have friends. Fortunately, we as a class did prove to be very supportive of each other and all became close. I wouldn’t say there has been anyone who would turn you away if you wanted to strike up a conversation. I feel very blessed by this and am so glad that I can sort of just float around friend groups in my class and feel like I belong in each one in a different way.”

Med Student #84 even described her peers as “family” and the deep connection she has with them through the shared experience of medical school:

“I have made friends that have become my family. They know me inside and out. They know how I think. They know how I act. What food I love and hate. What my habits are. They have seen me at my best and at my worst, and have always cheered me on at my best and at my worst. They have almost become my siblings. We bicker, fight, laugh, and push each other to do our best. And at the end of each day, I know that no matter what they will be there if I need something.”

Medical school affected their relationships in other positive ways as well. For example, Med Student #64 was grateful for meeting his girlfriend in medical school and being able to share their journey together and Med Student #15 even said he had become closer with his family because he had to make a concerted effort to maintain relationships given the time and energy demands of schoolwork. Nonetheless, the most significant source of social support seemed to be from their medical student peers, as students felt they were the only ones who could truly understand what each other were going through. In the quote above in which Med Student #84 said how her friends had become her family, she then went on to express how excited she was to one day have her peers as colleagues and the feeling that they all helped each other get to where they are. She even looks forward to her own children perhaps shadowing her future colleagues as medical students themselves and concluded that “I am very thankful that God placed each of them in my life because I know I wouldn’t be able to make it through this thing called “medical school” without them.”

## Discussion

### Utility of coping reservoir model

This analysis was a qualitative application of The Coping Reservoir Model of Medical Student Well-Being and, in particular, a test of the nature of the depleting and replenishing inputs set forth by Dunn et al. [22]. In general, the data were in alignment with the Coping Reservoir Model and the results provide support for medical student well-being as a dynamic process in which depleting and replenishing factors are constantly filling and draining students’ coping reservoirs. The depleting inputs found in this study are similar, but not identical, to those identified by Dunn et al. [22]. For example, our findings on students’ emphasis on the abundance of material and grades are consistent with prior research on the pre-clinical years [23]. Findings here are also in alignment with past research showing that students are often crippled with self-doubt in the form of imposter syndrome and other feelings of inadequacy [31]. Unlike the Coping Reservoir Model, however, we did not identify mentorship as a prevalent theme in first year students’ experiences. While not dismissing the great deal of stress that first year students face, this analysis emphasizes the role that positive experiences play in the pre-clinical medical school experience, which is typically characterized in the literature as a series of traumatic events that students need to overcome. Unlike other theories of coping in medical school, the Coping Reservoir Model provides a convenient and appropriate framework for avoiding the inclination to focus on the stress of the pre-clinical phase of medical school to the exclusion of the positive aspects.

### Making it ‘worth it’: human connection

This study suggests that what allows for optimism and hope to be as equally pervasive as negativity and self-doubt among medical students are the human connections they form with their medical student peers, patients, and, especially, the future patients that are idealized in their minds. These findings support prior research showing the importance of social relationships in medical school and how social support, especially from other medical students, buffers the adverse effects of stress on well-being [4, 32–36]. These findings are also consistent with other research that finds that 69% of first year students cite health care visits as their most rewarding experience, with approximately 84% of them feeling prepared for this early clinical exposure [37]. Findings here are also in alignment with other studies that show that the quality of the doctor-patient relationship is the strongest predictor of physicians’ work satisfaction [38, 39].

Results here that students begin medical school with a great deal of optimism are not surprising given that these findings are consistent with prior research [18, 21]. What this study contributes, however, is that the main source of the hope that it is going to get better is based on perceptions of the doctor-patient relationship that they will have once they start practicing medicine. Unfortunately, by all indications, this source of optimism, the idea that they will spend their days making meaningful connections with fellow humans, is a false hope. In fact, research shows that, compared to the first and second pre-clinical years, well-being gets worse in the third year of medical school, which is when students begin their clinical rotations and thus have a great deal of patient interaction [40, 41].

Although medical school is undoubtedly a time of great stress, we nonetheless assert that by labeling students’ reservoirs as containing ‘coping’ mechanisms, it denotes an inherently negative experience, and even trauma, with which needs to be dealt. The metaphor is not consistent with the findings of this study that show that inputs are both positive and negative and that what is potentially being drained is hope for the doctor-patient relationship. Thus, we suggest a shift in focus from a reservoir of coping to one of hope.

Research shows that students enter medical school with high levels of optimism, which is associated with better well-being [18, 21]. Future research should determine whether there is a ‘tipping point’ in which physicians’ *Hope Reservoirs* are completely drained by the realities of practicing medicine in a system that is generally not conducive to the development of long-term, meaningful connections with patients. That is, perhaps hope is drained due to factors such as doctors’ inabilities to spend sufficient time with patients or that their decision-making autonomy is compromised by the structure of the health care system, such as insurance company reimbursements and prior authorization policies. In other words, at what point, and due to what factors, do medical students, residents, and/or attending physicians conclude that it is no longer “worth it”? Or, conversely, as Mavor and colleagues suggest [33], and our Hope Reservoir concept supports, researchers could shift the focus from the risk factors of burnout to the social-psychological process that allows so many medical students, residents, and physicians to successfully remain optimistic in the face of such great stress.

### Limitations and contributions

The main limitation of this study is that the reflective writings were not anonymous, which may have influenced how free students felt to disclose personal vulnerabilities or criticisms about faculty/the medical school, especially given the literature, including the present study, that shows that medical students are consumed with insecurities. Nonetheless, there is no indication of students holding back in the content of these reflective writings. Students who were particularly nervous or shy likely did not provide consent for study participation and thus were selected out of the sample. Given that this was a small number of students, it is unlikely to have affected the results of this study. The second author (JS), who led the wellness course but does not grade them in any other course, anecdotally notes that students were very open in the small group discussions about their reflective writings, even about extremely personal matters and, further, students were reassured of the confidentially of both their writings and the in-class discussions. Although this study is a non-random sample of one medical school in the Midwestern United States, the students are for the most part representative of other pre-clinical medical students and thus we believe the findings here are generalizable.

It should also be noted that this study is limited in scope as it was not a full test of the coping reservoir model and cannot make causal claims regarding outcomes such as burnout and resiliency. Despite these limitations, this study makes four notable contributions to the literature on medical student well-being that outweigh its limitations. First, this study contributes to the limited research on the first year of medical school, which is knowledge that can be used to prevent the first signs of poor well-being. Second, by utilizing an interpretive description approach [28] to analyze reflective writings, students told stories in their own narratives, allowing for a rich analysis of their pre-clinical medical school experiences. Third, this study does not simply examine the stressors of medical school like most past studies tend to do, but rather it rather emphasizes the positive aspects of medical school that allow for many students to become physicians with high levels of well-being. Fourth, the main contribution of this study is that, while research has demonstrated that medical students have high levels of optimism [18, 21], this study identified the main source of that hope, which is idealized notions of building meaningful relationships with patients. Given that there is limited research on the positive aspects of the pre-clinical medical school experience, further research should replicate this analysis to confirm that social relationships—with patients and with each other— are the main factors that make medical school “worth it.”

### Implications for medical education to improve well-being

In addition to the theoretical contributions to The Coping Reservoir Model’s utility in understanding medical students’ well-being, the results of this study also have practical implications for medical education. As discussed above and illustrated in Fig. 1, the unifying theme across the various positive factors is that of social relationships—with patients and with each other. To that end, we propose the following two curricula implementations for improving well-being among pre-clinical medical students: first, to structure earlier, more, and longitudinal patient contact experiences into the curriculum and, second, to facilitate social solidarity amongst each other.

This study suggests that pre-clinical students would benefit from having more interactions with patients, as this study suggests that patient contact will foster hope that their stress will get better because it will make it worth it. This study shows that even half of a day once a month shadowing a preceptor (or occasionally volunteering or some other one-time experience) has a large and positive impact on students’ abilities to maintain hope. Thus, incorporating more clinical experiences into the pre-clinical years might have added benefit in mitigating the possible adverse effects of stress. Some medical schools have begun to depart from the strict historical preclinical/clinical divide, with there being some evidence that providing early clinical experiences has benefits for medical students. In a systematic review of 73 studies, Dornan and colleagues found a range of positive effects of early clinical experiences (e.g., clinical placements) for students, patients, and organizations, including building students’ confidence in interacting with patients [42]. Thus, increasing the frequency of earlier and more frequent patient contact might have the additional benefit of assuaging feelings of imposter syndrome and internal conflict about becoming a doctor, two of the negative inputs identified here and elsewhere [42]. Other studies similarly demonstrate the potential benefits of vertically integrating clinical experiences into the curriculum, such as the increasing implementation of longitudinal clerkships, student-run free clinics, and health care visits in a problem-based curriculum [37, 43, 44]. More research is needed to determine if there is a causal relationship between medical students’ well-being and exposure to patient care, particularly in the pre-clinical years since research consistently shows that clinical empathy sharply declines in the third year just as their patient contact is increasing [40, 41, 45]. Earlier and more exposure to patient care might reduce the shock of the “devil in the third year” that threatens their well-being [46] and give pre-clinical students more realistic expectations for future patient care.

In alignment with our finding that human connection makes medical school ‘worth it’ and the implication that it improves medical student well-being, this study suggests that additional efforts should be made to deemphasize competition and rather encourage cohesion among medical students, as this study finds that the pseudo-family relationships that the students form with each other are indispensable for their well-being. Reducing the high levels of competition surrounding grades found here as well as in other studies would also help alleviate some unnecessary stress that potentially turns the valuable resource of peer social support into a negative force and depletes from students’ hope reservoirs. This can be done with small efforts such as not publicizing class averages of exam scores. Another solution is to change the grading structure to pass/fail, as research shows that doing so improves well-being; studies show that the more the grade structure is stratified, the higher degree of burnout and, further, pass/fail grade structures increase social solidarity among medical students, as well as their well-being, while not compromising indicators of academic performance, including USMLE Step-1 scores [47, 48]. Slavin’s and colleagues’ study also provides evidence of the positive association of four curricular changes—pass/fail grade scheme, reduced contact hours, longitudinal electives, and learning communities— on mental health and social solidarity [49]. Another suggestion to decrease competition and rather increase social solidarity among medical student peers, medical schools could implement wellness courses that include the sharing of personal experiences like was required of the students in this sample. The results of this study support the proposal to shift the paradigm of pre-clinical medical student well-being from individual stress management and resiliency to how the structure of the curriculum can facilitate improved well-being.

## Conclusions: beyond burnout

Although the suggestion to increase patient contact early on could fuel students’ hope reservoirs in the pre-clinical years, this study also suggests that more long term structural solutions are needed in order for medical students to maintain the hope that keeps them going in the pre-clinical years. Medical schools have increasingly been implementing wellness electives into their curriculums, as the Liaison Committee on Medical Education requires schools to have a wellness program for accreditation [50]. On the one hand, such programs represent an appropriate shifting of focus to medical student well-being, as promoting health behaviors such as physical exercise [4] and mindfulness [51], which unequivocally help to alleviate stress among medical students. On the other hand, medical education reform tends to be a zero-sum game in which adding anything to the curriculum requires removing something else and so careful consideration needs to be given to what is included. For example, there is evidence that academic stressors adversely affect well-being significantly more so, above and beyond, personal stressors [52].

Placing the onus on individuals to improve their health and become more resilient is not likely to have the farthest scope possible in terms of increasing well-being. In fact, the widely-evoked terms in the literature of burnout and resiliency do not seem to be the best terminology to reflect medical student wellness, as they imply that both the cause and the responsibility is on the individual. Rather than the implication that medical students need to develop coping mechanisms to deal with the trauma of medical school, this study suggests that the best way to prevent setting pre-clinical medical students on a trajectory that eventually drains their hope reservoirs is to increase the positive aspects of medical school, which are the meaningful relationships they form with patients and with each other. With the minor adaptation of realigning the focus on hope, The Coping Reservoir Model helps to make this appropriate shift from the individual (i.e., burnout and resiliency) to the structure and culture of medical education (i.e., patient contact and reduced peer competition). In short, the goal should be to make medical school ‘worth it’ by making pre-clinical medical students’ false hopes about their future doctor-patient relationships not false.

## Data Availability

Data sharing is not applicable to this article.

## Abbreviations

UNMC: University of Nebraska Medical Center
LCME: Liaison Committee on Medical Education
SRQR: Standards for the Reporting Qualitative Research
